# Optimal minimum MU for intensity‐modulated proton therapy with pencil‐beam scanning proton beams

**DOI:** 10.1002/acm2.14435

**Published:** 2024-06-23

**Authors:** ByongYong Yi, Sina Mossahebi, Jenna Jatczak, Michelle Mundis, Thomas Houser, David Alicia, Dong Han, Rosette Gonzalez, Stephen Olis, Mark Zakhary

**Affiliations:** ^1^ Department of Radiation Oncology University of Maryland School of Medicine Baltimore Maryland USA; ^2^ Maryland Proton Treatment Center Baltimore Maryland USA; ^3^ New York Proton Center New York New York USA

**Keywords:** intensity‐modulated proton therapy, minimum monitor unit, proton pencil‐beam scanning, treatment time

## Abstract

**Purpose:**

A higher minimum monitor unit (minMU) for pencil‐beam scanning proton beams in intensity‐modulated proton therapy is preferred for more efficient delivery. However, plan quality may be compromised when the minMU is too large.

This study aimed to identify the optimal minMU (OminMU) to improve plan delivery efficiency while maintaining high plan quality.

**Methods:**

We utilized clinical plans including six anatomic sites (brain, head and neck, breast, lung, abdomen, and prostate) from 23 patients previously treated with the Varian ProBeam system. The minMU of each plan was increased from the current clinical minMU of 1.1 to 3–24 MU depending on the daily prescribed dose (DPD). The dosimetric parameters of the plans were evaluated for consistency against a 1.1‐minMU plan for target coverage as well as organs‐at‐risk dose sparing. DPD/minMU was defined as the ratio of DPD to minMU (cGy/MU) to find the OminMU by ensuring that dosimetric parameters did not differ by >1% compared to those of the 1.1‐minMU plan.

**Results:**

All plans up to 5 minMU showed no significant dose differences compared to the 1.1‐minMU plan. Plan qualities remained acceptable when DPD/minMU ≥35 cGy/MU. This suggests that the 35 cGy/MU criterion can be used as the OminMU, which implies that 5 MU is the OminMU for a conventional fraction dose of 180 cGy. Treatment times were decreased by an average of 32% (max 56%, min 7%) and by an average of 1.6 min when the minMU was increased from 1.1 to OminMU.

**Conclusion:**

A clinical guideline for OminMU has been established. The minMU can be increased by 1 MU for every 35 cGy of DPD without compromising plan quality for most cases analyzed in this study. Significant treatment time reduction of up to 56% was observed when the suggested OminMU is used.

## 1 INTRODUCTION

Pencil‐beam scanning (PBS) proton systems deliver programmed monitor units (MU) to each spot in each energy layer. Once the desired spot MU is reached, the beam control moves to the next spot position. Because of the time‐resolution limitation of the hardware, a small amount of dose may be delivered to a spot in excess of that for which it was programmed.^1^ Low‐weighted MU spots are the most challenging because of the relatively large contribution of measurement noise and statistical fluctuations. As the number of protons per spot decreases, PBS systems have a threshold below which spots become “undeliverable”, due to unacceptable spot delivery accuracy.^2^, ^3^ This lower limit for deliverable MU, referred to as the minimum MU (minMU) constraint of the delivery system, is one of the hardware constraints for PBS proton beams.^3^ This constraint creates a significant distortion of dose distribution from the ideally optimized distribution for intensity‐modulated proton therapy (IMPT) fields unless the minMU constraint is incorporated into the optimization process.^4^, ^5^ Two methods have been introduced to consider the minMU constraint in the process of IMPT optimization: (1) after optimization^4^, ^5^ or (2) as an optimization constraint.^6^, ^7^, ^8^, ^9^, ^10^, ^11^

Gao et al. recently demonstrated experimentally that the machine dose rate is proportional to minMU and that plan delivery can be even more efficient if a different minMU is assigned to each energy layer rather than assigning a global minMU per plan.^12^ Maximizing the minMU for stereotactic body radiation therapy using PBS is also useful because the treatment time is expected to be shortened due to increased dose rates.^11^ For similar reasons, a few reports have commented on the importance of minMU for proton arc and proton FLASH treatment.^13^, ^14^, ^15^, ^16^

The need for delivery efficiency in proton therapy has been emphasized.^17^ Finding the highest minMU while not compromising plan quality is an important task for routine IMPT planning. Commercially available treatment planning systems (TPSs), such as Eclipse (Varian Medical Systems; Palo Alto, CA)^18^ and RayStation (RaySearch Labs; Stockholm, Sweden),^19^ offer options for choosing the minMU in the plan optimization process.

Despite the importance of the optimized minMU (OminMU) in IMPT planning and the widely available tunability of minMU during IMPT optimization, no general guidelines for OminMU are available. This report systematically monitored changes in plan quality and delivery efficiency for different minMU to create a planning guideline for OminMU for IMPT using the ProBeam system (Varian Medical Systems; Palo Alto, CA). This report also developed a model of the treatment time reduction (TTR) per minMU, which explains the saturation of TTR when minMU exceeds a certain value.

## 2 MATERIALS AND METHODS

### 2.1 Patients and treatment planning

In our institutional review board–approved study, data were used from 23 patients previously treated to 6 anatomic sites (3 brain, 5 head and neck (HN), 5 breast, 2 lung, 3 abdomen, and 5 prostate cases). These cases were randomly selected, and their clinical IMPT plans were generated using 1.1 MU as the minMU in the RayStation TPS (V8–V11). Target volumes ranged from 8 to 2922 cc (median, 180 cc). Fractional and total doses were 110−600 cGy (median, 200 cGy) and 1800−7000 cGy (median, 5400 cGy), respectively (Table 1). Some of the plans such as brain cases, some breast cases, abdomen cases, and some prostate cases were planned with the single‐field optimization (SFO) technique, and the rest with multi‐field optimization (MFO).

**TABLE 1:** Characteristics of cases.

Case	Fx dose (cGy)	Rx dose (cGy)	Target volume (cc)	Treatment site/note	Number of fields per plan	Total MU
Brain1	600	1800	8	Partial brain/SRT	3	15,109
Brain2	110	5940	45	Partial brain/BID	4	6,848
Brain3	180	5400	85	Rt frontal	3	16,707
HN1	200	7000	64	Rt neck	2	15,866
HN2	210	6300	156	Bilateral head & neck/SIB, 170, 180, and 210 cGy/fx	4	80,656
HN3	200	5400	494	Bilateral head & neck	5	108,688
HN4	200	6600	307	Rt neck	3	64,577
HN5	210	6300	180	Lt neck/SIB, 200 and 210 cGy/fx	3	47,702
Breast1	200	5040	2922	Rt whole breast	2	220,799
Breast2	180	5040	2232	Rt whole breast	2	136,377
Breast3	300	4800	161	Lt partial breast	2	53,422
Breast4	180	5040	1720	Bilateral breast	3	247,504
Breast5	180	5040	1436	Rt whole breast	2	167,517
Lung1	200	7000	225	Lt lung	4	41,427
Lung2	200	6000	49	Rt lung	3	21,806
Abdomen1	387	5805	174	Liver/breath hold	2	56,223
Abdomen2	180	5400	47	Liver/breath hold	2	15,623
Abdomen3	360	5400	294	Pancreas/free breathing	3	52,871
Pelvis1	180	4500	493	Prostate/initial	3	66,262
Pelvis2	180	3420	48	Prostate/SFB	2	11,884
Pelvis3	180	4500	406	Prostate/initial	3	55,216
Pelvis4	180	3420	42	Prostate/SFB	2	9,564
Pelvis5	250	7000	500	Prostate/SIB, 180 and 250 cGy/Fx	4	66,182

Abbreviations: BID, twice per day; SFB, small‐field sequential boost; SIB, simultaneous integrated boost; SRT, stereotactic radiotherapy.

The same dose optimization objectives and constraints from the clinical plans (with minMU of 1.1 MU) were used when optimizing comparison plans with different minMUs. All other planning parameters such as range shifter, SFO/MFO planning, spot spacing, and energy layer spacing remained unchanged from the clinical plans. All plans were robustly optimized with 3 mm/3.5%, 5 mm/3.5%, or 5 mm/5%, depending on the anatomic site. The same robust parameters were applied for comparison plans, and plans were normalized to the same isodose level. Mean doses to organs at risk (OARs) and a few dosimetric parameters of target coverage, such as dose received by 1% of the volume (D1) and percentage of target volumes receiving 95% of the total prescription dose (PD) (V95) or 100% of PD (V100), were used for plan comparison. Maximum dose information was used for spinal cords and brainstem OARs in the analysis. Any plans showing >1% difference in any dosimetric parameter compared to the 1.1‐minMU plans were designated as not acceptable plans (NAPs).

### 2.2 Daily prescribed dose and minMU

DPD/minMU was defined as the ratio of the daily prescribed dose (DPD) to the minMU (cGy/MU).

(1) $$\frac{\mathrm{DPD}}{\mathrm{minMU}}=\frac{\mathrm{Daily}\;\mathrm{prescribed}\;\mathrm{dose}\;\left(\mathrm{cGy}\right)}{\mathrm{minMU}\;\left(\mathrm{MU}\right)}.$$

The DPD/minMUs of each comparison plan were chosen as >40 (small minMU group or S group), 30−40 (medium minMU group or M group), and <30 cGy/MU (large minMU group or L group). From the DPD/minMU, corresponding minMUs were determined. The minMUs of the S, M, and L groups for a conventional fraction size of 180 cGy per fraction corresponded to 3, 5, and 7, respectively. The DPD/minMU ranges for each group were determined semi‐empirically based on preliminary testing of TTR of a few conventional fraction cases with a variety of minMU values.

### 2.3 Beam delivery efficiency model

Beam delivery efficiency was evaluated by measuring the treatment times of each plan with different minMUs. The ProBeam proton system (Varian, Palo Alto, CA) was used in developing the TTR model.

TTR is expressed as a function of the ratio of the total treatment delivery time (TTT) for 1.1‐minMU, $\mathrm{TT}T_{1.1}$, to that for a higher minMU, $\mathrm{TT}T_{\mathrm{minMU}}$:

(2) $$\mathrm{TTR}\;\left(\mathrm{minMU}\right)=100\times \left(\frac{\mathrm{TT}T_{1.1}}{\mathrm{TT}T_{\mathrm{minMU}}}-1\right)\left(\%\right)$$

TTT is the sum of the spot delivery time, SDT, the spot switching time, SST, and the energy layer switching time, EST:

(3) TTT=SDT+SST+EST.

SDT is a function of the monitor units $MU_{E}$ for each energy layer, and the dose rate (DR) of each energy layer. The dose rate is a function of energy and minMU:

(4) $$\mathrm{SDT}=\sum_{E}\frac{MU_{E}}{\mathrm{DR}\left(E,\;\mathrm{minMU}\right)}\;.$$

To simplify the model, it is assumed that the MU of each energy layer is the same. Then, Equation (4) becomes:

(5) $$\mathrm{SDT}=\frac{MU_{\mathrm{Total}}}{N_{E}}\;\sum_{E}\frac{1}{\mathrm{DR}\left(E,\;\mathrm{minMU}\right)},$$

where, $MU_{\mathrm{Total}}$ is the total number of monitor units and $N_{E}$ is the number of energy layers.

An approximate dose rate model is designed based on fig. 2 from Gao et al., showing dose rate measurements for different dose rates.^12^ Their observation is that dose rate increases to about 2100 MU/min when the minimum MU increases above 1 MU:

(6) $$\begin{array}{c} \mathrm{DR}\;(\;E,\mathrm{minMU})=k\times \mu \times minMU,\;\mathrm{if}\;min\mathrm{MU}\leq minM\\ \;U_{threshold}=k\times \mu \times minMU_{threshold},\\ \;\mathrm{if}\;min\mathrm{MU}\geq minMU_{threshold} \end{array}$$

where *μ* is 2100 MU/min, and the dose rate correction parameter k is 1.0.

Another finding from Gao et al. is that the dose rate saturates when minMU becomes greater than 2 for 70 MeV and the saturation point increases 1.15 MU for every 10 MeV proton energy.^12^ This can be expressed as:

(7) $$\;minMU_{\mathrm{threshold}}=2+1.15\times \left(E-70\right)/10,$$

where *E* is the proton energy in MeV.

Since SST in Equation (3) is known to be short, it is ignored.

Therefore, EST can be expressed as:

(8) $$\mathrm{EST}\;=\;\delta \times N_{E},$$

where δ is the energy switching time, assumed to be 0.8 s in this report.^13^

From Equations (3) and (8), the total treatment delivery time is given as:

(9) $$\begin{array}{ccc} \mathrm{TTT} & = & SDT+\mathrm{SST}+EST,\\ & = & \frac{MU_{Total}}{N_{E}}\sum_{E}\frac{1}{DR_{E}}+\mathrm{SST}+\delta \times N_{E},\\ & = & \frac{MU_{Total}}{N_{E}}\sum_{E}\frac{1}{DR_{E}}+\delta \times N_{E}, \end{array}$$

The treatment time reduction from Equation (2) can be rewritten as:

(10) $$\begin{array}{ccc} & & \mathrm{TTR}\;\left(\mathrm{minMU}\right)\\ & = & \;100\times \left\{\frac{\left(\frac{MU_{\mathrm{Total}}}{N_{E}}\sum_{E}\frac{1}{DR_{E}\left(E,\;1.1\right)}\;+\;\delta \;\times \;N_{E}\right)}{\left(\frac{MU_{\mathrm{Total}}}{N_{E}}\sum_{E}\frac{1}{DR_{E}\left(E,\;\mathrm{minMU}\right)}\;+\;\delta \;\times \;N_{E}\right)}-1\;\right\} \end{array}$$

TTRs of the plans with different minMUs relative to those of minMU 1.1 were measured for all of the cases and the results were compared to the model, Equation (10). All plans were delivered to determine TTT. TTT was acquired from the treatment record system (Aria, Varian, Palo Alto, CA) after the treatment delivery. Patient setup or gantry motion is not included in TTT.

## 3 RESULTS

**FIGURE 1:**
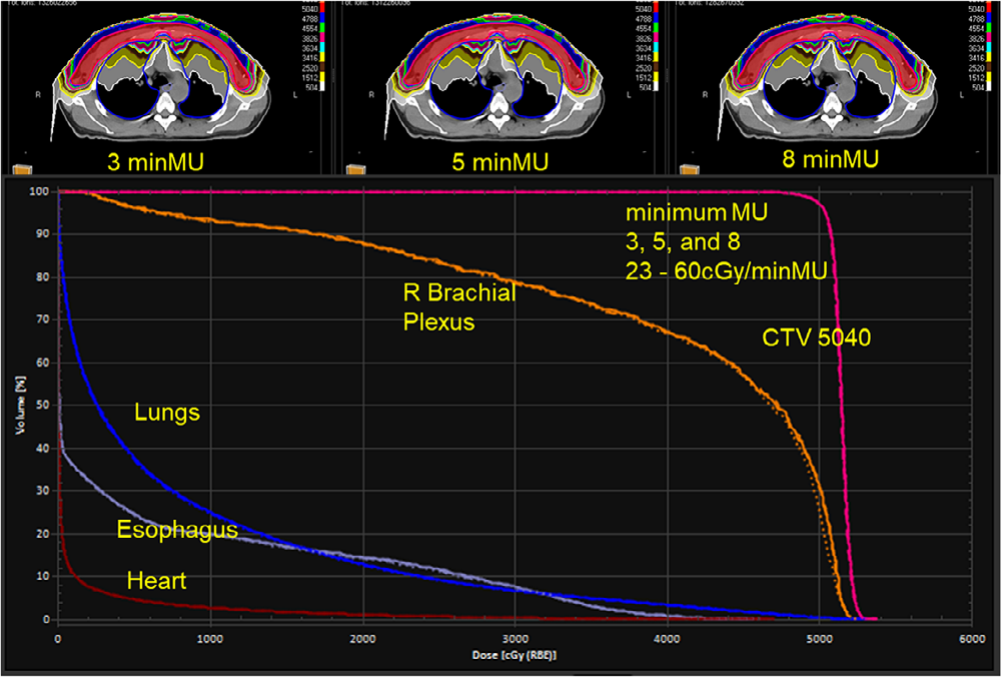
Dose distributions and dose–volume histograms of minimum MU constraints (3, 5, and 8 MU) for Breast 1 case. Solid, dashed, and dotted lines are for 3, 5, and 8 MU plans, respectively. Little dose differences between plans are observed for different minimum MUs. All three plans are acceptable.

**FIGURE 2:**
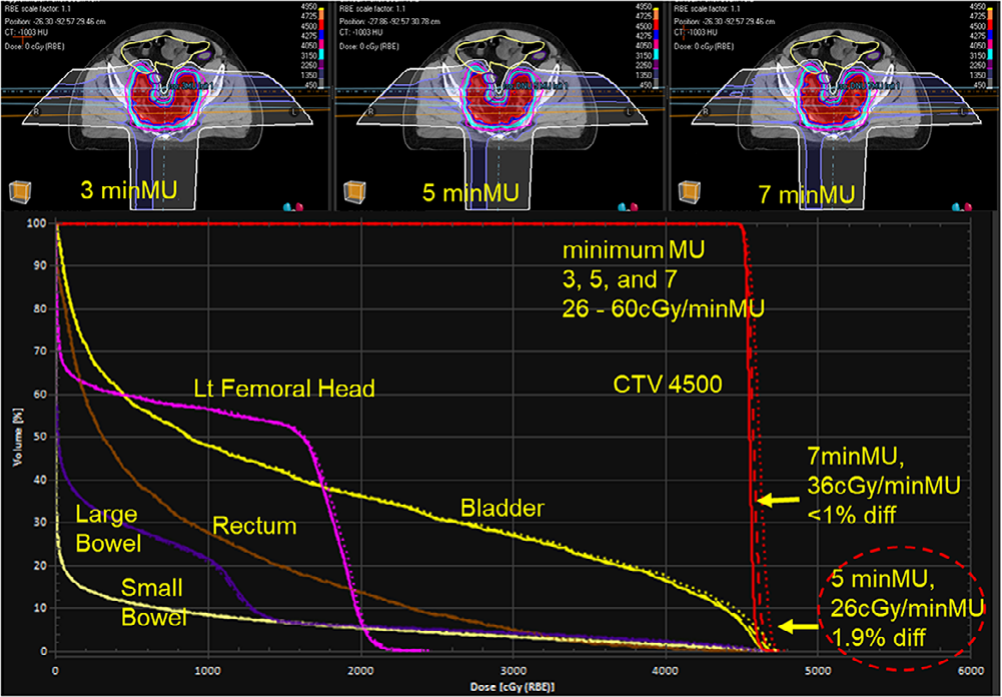
Dose distributions and dose–volume histograms of minimum MU constraints (3, 5, and 7 MU) for Pelvis1 case. Solid, dashed, and dotted lines are for 3, 5, and 7 MU plans, respectively. The 7‐minMU plan is a not acceptable plan (NAP) since D1 of CTV differs >1% than that of the 1.1 MU plan. This case is one of six NAP plans in this study (see Table 2).

**FIGURE 3:**
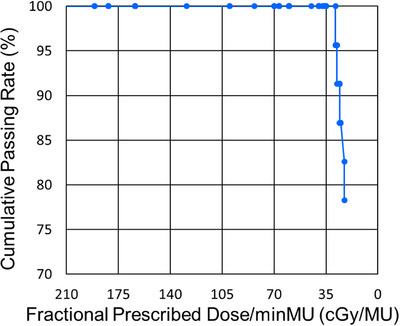
Plan passing rate versus DPD/minMU. Plan passing rate was defined as the ratio of the number of cases with target D1 difference >1% not acceptable plan (NAP) to the total number of cases (23 cases). The passing rate starts to drop when DPD/minMU becomes less than 30.

Among the dosimetric parameters, target D1 was the most sensitive and could change >1% when minMU exceeded its optimal value. Other parameters demonstrated <1% differences between the 1.1‐min plan and all S, M, and L plans. Therefore, target D1 was used to determine NAP status for different minMUs. Figure 1 shows representative dose–volume histograms (DVHs) of the plans with minMUs of 3, 5, and 7 MU for the Breast1 case (the DVHs of the 1.1‐minMU plans are not shown in Figures 1 and 2, because the curves almost overlap those of the 3‐minMU plans). Little variation in DVHs for all dosimetric parameters was found between the 3‐minMU plan and those of different minMUs. However, the target D1 of the 7‐minMU plan showed >1% difference compared to the 3‐minMU plan for the Pelvis1 case (Figure 2), making this 7‐minMU plan a NAP. Figure 3 shows the plan passing rate per DPD/minMU, indicating no significant changes in plan quality for DPD/minMU of ≥35 cGy/MU. The plan passing rate was defined as the ratio of the number of NAP cases to the total number of cases (23 cases). Table 2 shows the plan quality degradation with minMU change. As long as DPD/minMU ≥35 cGy/MU, a 100% passing rate could be achieved. A DPD/minMU of 35 corresponded to 5 minMU for the fraction dose of 180 cGy. The passing rate began to decrease quickly when DPD/minMU ≤30 cGy/MU (Figure 3). Based on the data presented in Figure 3 and Table 2, it is suggested that a DPD/minMU of 35 cGy/MU is the optimized value OminMU. For the CTV V95 metric, the average of the worst‐case robustness scenario was 96.9 ± 0.04 and 96.9 ± 0.03 (*p* = 0.97) for 1.1 minMU and 5 minMU plans, respectively.

**TABLE 2:** Minimum MUs and resulting reductions in number of spots and treatment times.

	S^a^		M^a^		L^a^		Change in %		Reduction in %	
Cases	minMU	PD/ minMU	minMU	PD/ minMU	minMU	PD/ minMU	MU^b^	Number of energy layers^b^	Number of spots^c^	Total treatment delivery time^c^
Brain1	10	60	17	35	24	25^d^	0	4	70	30
Brain2	1.1	100	3	37	4	27^d^	1	4	63	7
Brain3	3	60	5	36	7	26	−1	0	66	13
HN1	3	67	5	40	8	25	0	4	21	24
HN2	3	70	5	35	8	26	−1	2	37	29
HN3	3	67	6	40	8	25	0	2	30	33
HN4	3	67	6	40	8	25	0	0	44	36
HN5	3	70	5	35	8	26	0	2	53	28
Breast1	3	67	6	40	8	25	−4	−4	28	40
Breast2	3	60	5	36	7	26	−1	0	48	44
Breast3	3	100	8	38	12	25	−1	0	27	17
Breast4	3	60	5	36	8	23	0	0	43	40
Breast5	3	60	5	36	7	26	1	−1	78	41
Lung1	3	67	5	40	7	29	0	1	68	31
Lung2	3	67	5	40	7	29^d^	1	0	21	16
Abd1	3	129	11	35	14	28	0	0	69	35
Abd2	3	60	5	36	8	23^d^	−2	0	25	38
Abd3	8	60	10	36	14	26	2	0	40	46
Pelvis1	3	60	5	36	7	26^d^	1	0	52	42
Pelvis2	3	60	5	36	7	26	−1	0	39	26
Pelvis3	3	60	5	36	7	26	0	0	40	38
Pelvis4	3	60	5	36	7	26	1	0	33	26
Pelvis5	3	83	5	36	10	25	2	3	49	56

^a^ S minMU corresponded to DPD/minMU > 40 (3 minMU for a 180cGy/fx case); M minMU corresponded to DPD/minMU of 30−40 (5 minMU for a 180cGy/fx case); L minMU corresponded to DPD/minMU < 30 (7 minMU for a 180cGy/fx case).

^b^ Change of number from 1.1‐minMU plan to M minMU plan.

^c^ Reduction of number from 1.1‐minMU plan to M minMU plan.

^d^ Not Acceptable Plan, that is, plan quality degradation since change of target D1 > 1%.

Table 2 shows a 21%−78% decrease in the total number of spots and an average 32% (range 7%−56%) decrease in treatment delivery time (1.6 min shorter on average) for the M plan group relative to the 1.1‐ minMU plan group. Despite decreases both in number of spots and treatment delivery time, the two parameters are not correlated (correlation coefficient <0.05). Unlike the above two parameters, the total MU and the number of energy layers remained relatively unchanged within 6%.

**FIGURE 4:**
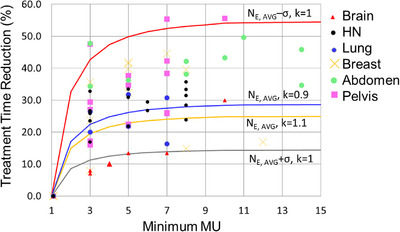
Treatment time reduction (TTR) versus minMU. Solid curves from Equation (10) developed in this study and the actual measurements (black dots). Variation of TTR is subtle when ±10% change of the dose rate constant (blue and yellow curves). By contrast, TTR changes dramatically when the number of energy layers changes from *N*_E_, _AVG_ − *σ* (orange curve) to *N*_E_, _AVG_ + *σ* (grey curve), where *N*_E_, _AVG_ is average number of energy layers and *σ* is the standard deviation of number of energy distributions of 23 cases. Majority of the measured TTRs are within ± *σ* curves.

Figure 4 demonstrates TTR per minMU (solid curves) of the model developed in this study using Equation (10) and the actual TTR measurements (black dots). Orange and grey curves are the calculated TTR for *N*_E_, _AVG—_ *σ* and *N*_E_, _AVG_ + *σ*, respectively, where *N*_E_, _AVG_ is the average number of energy layers and *σ* is the standard deviation of the number of energy layers for the 23 cases investigated in this study. More than 90% of measured TOR points are within these two *N*_E_, _AVG_ ± *σ* curves. When dose rate is changed within ± 10%, corresponding to variation in the dose rate constant *k* of Equation (6) between 0.9 and 1.1 (blue and yellow curves in Figure 4), the Ter variation does not change much (<5%). TTR starts to saturate when minMU ≥ 10 for both the measurements and the model. Little TTR benefit is found for minMU > 15. The rate of TTR increase becomes <0.5% per unit of minMU increase.

## 4 DISCUSSION

Our analysis of 23 cases showed that the total MUs and total number of energy layers of a plan do not change significantly when the minMU changes. Variations in both parameters were within 6% (average, <0.1%) of those from 1.1‐minMU plans, regardless of minMU. On the other hand, the total number of spots and the treatment time decreased dramatically when minMU increased. Howard et al.^2^ found that plan quality degraded rapidly when minMU increased and spot spacing remained fixed at 4 mm. However, less degradation was observed when spot spacing was increased to 8 mm.^2^ Wider spot spacing was required to maintain the plan quality when minMU increased, which is consistent with our finding: decreased number of spots for increased minMU plans. Zhu et al. reviewed the dependence of treatment delivery efficiency on spot spacing and energy layers. They concluded that the number of spots is not a major determining factor in treatment time, whereas the number of energy layers is.^13^ Spot size is also affecting the quality of plan because different spot size requires different spot spacing to optimize the dose distribution.^20^ The range of proton energies used in this study is from 70 to 220 MeV. Spot sizes of 70, 110, 150, 190, and 230 MeV proton beams are 6.0, 4.9, 4.5, 4.2, and 4.1 mm. The different spot sizes used for the cases in this study may be one of the contributing factors for different patterns of the reduction of the number of spots. In this study, beam delivery times (Table 2) showed noticeable reductions when minMU was increased, despite the fact that the number of energy layers did not change. Considering this, it appears that the main source of improved treatment delivery efficiency is the increased dose rate due to increased minMU, which is consistent with the finding of Gao et al.^12^

This study identifies D1 as sensitive across various minMU plans. One potential explanation on why D1 might be particularly sensitive to minMU variations could be the trade‐off between dose uniformity and modulation with increasing minMU. This might lead to more hot and cold spots within the target and as the normalization isodose lines are the same, it may primarily impact D1. Further, since all the spots are in or close to the target volume, the impact may be less in other critical structures outside the target.

Selection of the 1% DVH deviation threshold for NAPs was determined by expert consensus at our institution. Two experienced clinical physicists, dosimetrists, and physicians participated in the determination process. Five NAPs were identified in the L set of plans: 2 brain cases and 3 non‐brain cases. Two out of three brain cases demonstrated plan quality degradation as minMU increases in the L group. The maximum MU limit constraints for brain IMPT may contribute to this tendency. Maximum spot MU for all brain cases based on our institutional treatment planning guidelines is limited to 35 MU in order to reduce the risk of toxicity from heavily weighted spots in the brain.^21^ For the case of Brain1, the L plan had a minMU of 24 MU. Therefore, a minimal beam modulation was expected considering the limited spot weighting range (maximum, 35 MU; minimum, 24 MU). As a result, more significant dosimetric impact is unsurprising compared to cases with higher maximum MU and less restricted modulation. Excluding brain cases which have a maximum dose limitation, 2 of 11 SFO plans and 1 of 9 MFO plans demonstrated NAPs in the L group. No trends related to SFO or MFO planning strategies are found on the plan quality degradation with increased minMU.

Treatment time reduction with increasing minMU is significant, up to 56% as shown in Table 2. Figure 4 demonstrates TTR change with minMU. The actual dose rate of the machine may differ by site. The TTR changes when the dose rate slope (2100 MU/minMU/s) of Equations (6) and (7) changes. As shown in Figure 4, the TTR variation for ± 10% of dose rate change is relatively subtle. The majority of measured TTR points are within these two *N*_E_, _AVG_ ± *σ* curves. This shows Equation (10) delivered in this study predicts the TTR reasonably well. TTR saturation, however, happens at the same minMU range, regardless of dose rate and number of energy layers. The source of the saturation is $\mathrm{minM}U_{\mathrm{threshold}}$ in Equation (6). Because the reduction of SDT in Equation (10) saturates due to $\mathrm{minM}U_{\mathrm{threshold}}$, the dose rate ratio DR(E,minMU) also saturates when the minMU reaches $\mathrm{minM}U_{\mathrm{threshold}}$. The number of energy layers $N_{E}$, plays a role in determining the TTR value in Equations (9) and (10). If EST > > SDT, the TTR value will be lower since decreased SDT plays less of a role in determining TTR, while higher TTR is expected when EST < < SDT. It is noted that reducing the number of energies (*N*_E_) will reduce the treatment time further. $N_{E}$ showed a wide range (20–192) in the plans used in this report primarily due to the fact that the treatment volumes have a wide range (8–2922 cc).

EST is chosen as 0.8 s for the ProBeam system used in this study,^13^ but different vendors and accelerator systems may have different EST.^21^ SST is ignored in Equations (9) and (10). Poulsen et al. determined the scanning speed of ProBeam machine to be 6.92 mm/ms and 32.1 mm/ms for *x* and *y* axis, respectively.^22^ Shen et al.^23^ reported 5.9 mm/ms and 19.3 mm/ms for Hitachi machine (ProBeat V5, Hitachi, Japan). Considering such fast speeds, Equation (10) is still valid if SST is ignored.

The definition of monitor unit is not necessarily the same among different proton machines. Gillin et al. defined 217 MU of Hitachi delivery System to be: A 217 cGy uniform dose delivered to a 1‐L volume of water centered at the isocenter using pencil beams with the proton range of 21.0–30.6 g/cm^2^, a nominal SOBP width of 10 cm, and a 10 × 10 cm^2^ field size.^24^ By contrast, IBA defines 1MU to correspond to 3 nC collected in a 1 cm air‐filled ion chamber,^25^ and Varian defines 1 MU as the smallest charge that can be assigned to a spot. Applying the minimum MU guideline suggested in this report to different institutions and machines requires some effort, including testing of plan quality degradation and evaluation of TTR. To test the plan quality degradation, it is not necessary to repeat every test performed in this study. Equation (10) is still valid for different machines. The solid lines of Figure 4 using Equation (10) can be generated for different proton machines to find where the saturation starts. Then, plan degradation tests per minMU increase can be performed for a few cases. The minMU which starts TTR saturation is the OminMU. Since it is in the saturation region, OminMU does not need to be accurate. Also, no plan quality changes were found for M plans and only a few plan quality degradation cases are found for L plans. This suggests that OminMU can be found using a few cases for different machines.

One of the two limitations of this paper is that the OminMU is optimization algorithm dependent. A better algorithm than used in this study may generate higher OminMU. Zhu et al.^11^ demonstrated higher OminMU can be achieved for IMPT, SBRT, proton arc, and FLASH using their algorithm. Zhu et al., however, did not compare TTR per different minMUs. Also, the paper did not suggest any rationale for the use of minMU beyond the saturation point. Another limitation is that the model uses simplified energy distribution assumption. Equation (5) assumes that MUs are evenly distributed to each energy layer. Depending on the energy distribution, the TTR saturation point may be changed. Despite the simplified model, the measured TTR matches well with the model as shown in Figure 4. This report suggests DPD/minMU of 35 cGy/MU as the OminMU. More studies on potential benefits of using higher OminMU > 10MU, such as fewer spot position errors, are needed.

## 5 CONCLUSIONS

Patterns of plan quality degradation resulting from increased minMU were conducted. Among the dosimetric parameters, target D1 was the most sensitive dosimetric parameter and changed noticeably when minMU varied. For the M minMU plan group or DPD/minMU of 35 cGy/MU, none of 23 cases showed a D1 exceeding 1% compared to the 1.1‐minMU plan. Six cases among 23 cases for the L minMU plan group showed such a difference. The established OminMU as a planning guideline was determined to be 1 MU per every 35 cGy of DPD without compromising plan quality, with an accompanying reduction in treatment delivery time of 32% compared to the 1.1‐minMU plan group. This paper also demonstrated that TTR saturates when minMU reaches greater than 10 MU. The TTR model developed in this study also shows that little TTR benefit is found for minMU > 10, consistent with measurements.

## AUTHOR CONTRIBUTIONS

The authors confirm contributions to the paper as follows: *Study conception and design*: ByongYong Yi, Sina Mossahebi, and Mark Zakhary. *Data acquisition*: ByongYong Yi, Sina Mossahebi, Mark Zakhary, Dong Han, Jenna Jatczak, Michelle Mundis, Thomas Houser, David Alicia, Rosette Gonzales, and Stephen Olis. *Analysis and interpretation of results*: ByongYong Yi, Sina Mossahebi, and Mark Zakhary. *Final approval of the version to be published*: All authors reviewed the results and approved the final version of the manuscript.

## CONFLICT OF INTEREST STATEMENT

The authors declare no conflicts of interest.

## Data Availability

Data that support the findings of this study are available from the corresponding author upon reasonable request.
